# The Amalgamation of Dublin Medical Schools

**Published:** 1888-09

**Authors:** 


### THE AMALGAMATION OF DUBLIN MEDICAL SCHOOLS.

The scheme of amalgamation which we alluded to last week has been finally revised by the Special Committee of the Council of the College of Surgeons and has occupied the Council for a day's discussion. We understand that very satisfactory progress has been made with it, and that its promotors are saguine that it will shortly receive the *imprimatur* of the Fellows of the College and of the Lord Lieutenant, whose function it will be to approve the needful changes in the College by-laws. If the progress of the scheme is undisturbed, the Carmichael and Ledwich Schools will cease to exist as separate institutions, and will work in conjunction with the School of the College from and after next session, but it will not be possible to arrange for the accommodation of the whole of the students on the College premises, and therefore the Carmichael College will remain open for certain departments of study until such accommodation can be provided.—*Medical Press and Circular*.

